# Hydrogels in Contraceptive Applications and Their Mechanisms of Action

**DOI:** 10.1021/acsabm.5c00556

**Published:** 2025-06-04

**Authors:** Kyla M Raoult, Bert Klumperman

**Affiliations:** Department of Chemistry and Polymer Science, 26697 Stellenbosch University, Private Bag X1, Matieland, Stellenbosch 7602, South Africa

**Keywords:** contraception, hydrogels, gender inequality, mechanism of action, biocompatibility, bioengineering

## Abstract

The prevention of unplanned pregnancies is a global problem that requires immediate attention. The livelihoods of both men and women are affected by this issue. The best way to prevent unplanned pregnancies is by means of contraception. The majority of contraceptive options currently available target female contraception, with men’s options mostly limited to condoms and vasectomies. A relatively unexplored solution to this problem is the use of hydrogels in contraceptive applications. Hydrogels are water-insoluble polymer networks that have the ability to absorb water and, once swollen, start to mimic the structure and flexibility of natural tissue. Hydrogels have been used in a multitude of biomedical applications for this reason. The purpose of this review is to explore some of the hydrogel systems that have been designed for this purpose, placing emphasis on the mechanisms of action that provide contraception. Understanding the mechanisms of action behind hydrogel contraceptives allows for the design and development of new methods and the improvement of existing formulations. The majority of the hydrogels that have been designed for contraceptive purposes again target the child-bearing half of the couple; however, this growing field of research is relatively new and unexplored, and some of the technologies that are currently being investigated might soon find their way to the market. The use of hydrogels in contraceptive applications is a promising solution to this global issue.

## Graphical abstract

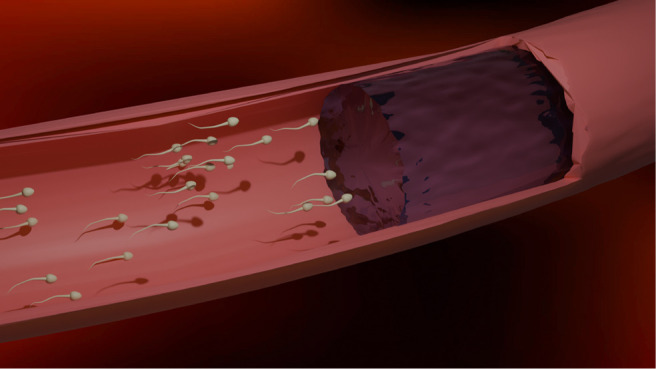

## Contraception

Many aspects of human well-being are related to the extent to which one can control and plan one’s life. Unplanned and often unwanted pregnancies can have a significant effect on spendable income, education, health, etc. An increased availability of contraceptives for both men and women will lead to an increased opportunity for people to plan their lives and focus on their health and well-being before acquiring the responsibilities of parenthood.^1^

Family planning can lead to a decrease in one’s personal financial strain, decreasing the likelihood of individual poverty by eliminating the need to provide for additional, unplanned family members. By decreasing a country’s population, the extent of countrywide poverty could also potentially be reduced.^2^

The increased availability of contraception is likely to lead to a decrease in the population size worldwide. If this is the case, this will reduce all disadvantages and negative effects that are associated with the exponentially increasing population growth, such as environmental degradation, limited resource availability, etc.

As women are naturally the most affected by unplanned pregnancies, the issue strongly contributes to gender inequality. The availability of a desirable and efficient male contraceptive will allow men to contribute to the prevention of unplanned pregnancies, reducing this inequality and improving the lives of both men and women. Contraception has been around for decades and has naturally targeted the child-bearing half of couples. There has, however, been an increased interest among men in contributing to the prevention of unwanted pregnancies.^3,4^

The purpose of this comprehensive but not exhaustive literature review is to explore the potential of hydrogel technologies for contraceptive applications and to identify their underlying mechanisms of action.

## Current Approaches to Contraception

The oldest known method of contraception is likely “coitus interruptus”, a natural method relying on the male partner withdrawing his penis from the vagina prior to ejaculation.^2^ Since then, researchers have made significant advancements from this method toward more reliable methods including barrier methods (male and female condoms), intrauterine devices (IUDs), hormonal contraception, and sterilization (Figure 1).^2^

**Figure 1:**
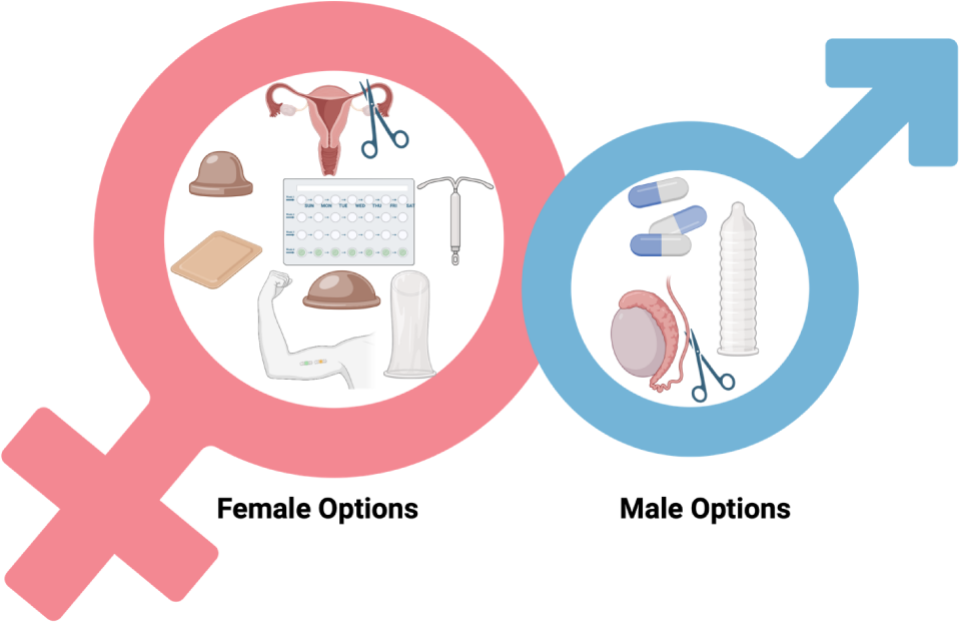
Some of the currently available contraceptive options for females (left) and males (right) (image created by using BioRender).

According to the World Health Organization, condoms have been reported to have a 13% fail rate. IUDs are not accepted by all bodies and can cause discomfort. Hormonal contraceptives often lead to negative side effects, and sterilization, such as vasectomy or tubal ligation, is, in most cases, a permanent procedure. In addition to these limitations, the majority of contraceptives currently available target female contraception, as can be seen by the large number of examples provided for females in Figure 1, versus the limited examples available for males. Much discussion has already taken place about the limitations of the currently available methods, and improvements to these limitations may lead to an increased use of contraceptives due to user satisfaction and may result in the reduction of unintended pregnancies.^5^

Improvements could include the reduction of side effects of hormonal methods and making these methods more user-friendly, reliable, comfortable, and confidential.^5^ In an attempt to achieve these improvements, researchers have started focusing on nonhormonal options and/or noninvasive approaches. Hydrogels may prove to be a promising solution.

## Hydrogels

Hydrogels are water-insoluble polymer networks with the ability to absorb vast amounts of bodily (aqueous) fluids. These polymer networks were first developed as hydrogels by Wichterle and Lim in 1960, whereby they were described as structures that can contain a particular amount of water, are biologically compatible, and are permeable to metabolites.^6^ In order to have these properties, the hydrogels must have hydrophilic moieties present in their three-dimensional structure that become hydrated in aqueous media.^6–9^ They must be three-dimensional and cross-linked in such a way as to prevent dissolution.^6^ Due to their large water content, hydrogels are highly flexible, similar to natural tissue.^9^ The volume of the hydrogel is determined by its water content, which should remain constant at equilibrium, and is dependent on the structure of the polymers used, as well as the number of cross-links.^6^ The first clinical application of hydrogels was that of soft contact lenses.^6^ Nowadays, hydrogels find themselves in numerous applications, including but not limited to agriculture (soil conditioning and controlled release of fertilizers), environmental applications (water purification and pollutant adsorption), diapers, and personal care products (Figure 2). These applications have been extensively reviewed, including reviews by Ullah et al. and Thakur et al.^10,11^

**Figure 2:**
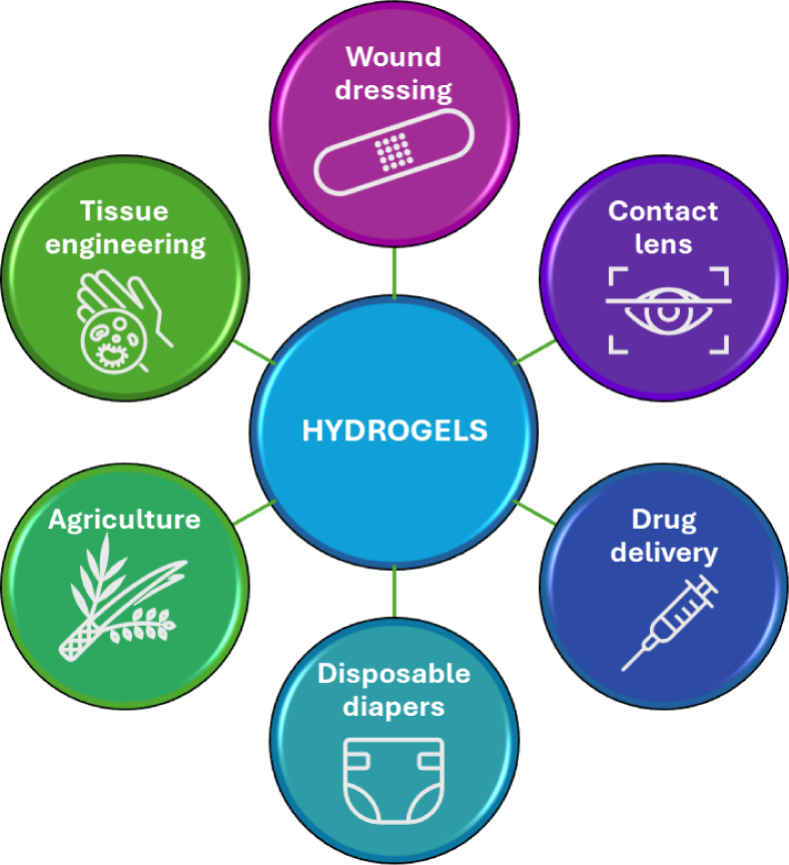
Some Applications of Hydrogels.

As has recently been reviewed by Chamkouri et al., hydrogels have gained significant interest in various biomedical applications (drug delivery, tissue engineering, and wound healing) because of their soft structure, water absorption ability, resemblance to the extracellular matrix (ECM), and their biocompatibility.^8^ Injectable hydrogels intended for medical applications must be biocompatible, nontoxic, stable, and biodegradable, and have good viscosity, as well as suitable mechanical properties, among other essential properties.^8,12^ Furthermore, the hydrogel structure must be compatible with cells, tissues, and body fluids.^12,13^ Upon degradation, it is important that degradation products are also biocompatible and have a low toxic potential.^7^

## Classification of Hydrogels^10,11^

The classification of hydrogels has been extensively studied and reviewed. Hydrogels can be classified based on their origin, namely, natural, synthetic, and hybrid hydrogels. These hydrogels are derived from natural, synthetic, and a combination of natural and synthetic polymers, respectively. Natural hydrogels, made from natural polymers such as cellulose or chitosan, are nontoxic, biocompatible, and biodegradable, whereas synthetic hydrogels, made from synthetic polymers such as poly­(ethylene glycol) (PEG), have the advantages of increased ability to absorb water, longer life, and increased gel strength.^8^ The biocompatibility of these synthetic hydrogels is determined by the polymer units used to form the hydrogel.

Hydrogels can be classified based on composition, namely, homopolymeric, copolymeric, and multipolymer interpenetrating polymeric hydrogels. Hydrogels can be classified based on ionic charge, namely neutral, ionic, and ampholytic hydrogels, which carry no charges, cationic or anionic charges, or both types of charges, respectively. Cationic hydrogels show increased swelling at lower *p*H, whereas anionic hydrogels show increased swelling at higher *p*H. Hydrogels can be classified in terms of porosity, namely nonporous, microporous, and superporous hydrogels. Hydrogels can be classified according to their physical structures and chemical composition. Here, they are divided into amorphous (or noncrystalline) and semicrystalline hydrogels, whereby amorphous hydrogels are characterized by randomly distributed polymer chains, and semicrystalline hydrogels are characterized by a combination of amorphous and crystalline regions within the gel.

Furthermore, hydrogels can be classified based on the type of external stimulus (or stimuli) they are capable of responding to.^14^ Stimuli-responsive hydrogels can be classified based on their response mechanism to various external stimuli. External stimuli could include physicochemical stimuli such as *p*H (*p*H-responsive hydrogels), temperature (thermoresponsive hydrogels), light (photoresponsive hydrogels), and electric and magnetic fields (electro- and magneto-responsive hydrogels), as well as other stimuli such as ultrasound irradiation, redox reactions, or the presence of enzymes, metals, small molecules, and proteins (entity-responsive hydrogels). Some hydrogels can undergo changes when exposed to multiple external stimuli (multistimuli-responsive hydrogels).

Finally, hydrogels can be classified based on their mechanism of cross-linking, namely, physical and chemical cross-linking. The presence of cross-links is required for the network to remain intact. Physical cross-linking can include entanglements, ionic interactions, and hydrogen bonding, whereas chemical cross-linking involves the formation of covalent bonds between cross-linked polymers.^8,9^ Chemically cross-linked hydrogels may be advantageous as they possess improved stability over physically cross-linked hydrogels and may provide better mechanical strength; however, this is not always the case as both physically and chemically cross-linked hydrogels span a wide range of different properties.^7,8^ To compete in biocompatibility with physical hydrogels, toxic cross-linking agents must be avoided in the synthesis of chemical hydrogels.

Chemical cross-linking involves various reactions such as photochemical cross-linking, enzymatic reactions, and click reactions, whereas physical cross-linking involves modification of intramolecular forces including electrostatic forces, hydrogen bonding, and hydrophobic interactions by the use of ionic cross-linking, temperature-dependent methods, and *p*H-dependent methods, among others. A summary of these reactions and interactions can be found in the reviews by Akhtar et al. and Chamkouri et al.^7,8^ The advantages of some of the biocompatible reactions and mechanisms are briefly summarized in Table 1. As these reactions are biocompatible, they could be used for cross-linking hydrogels in situ, as well as for hydrogels that are preformed outside the body and then applied, post-formation, to a specific application. The cross-linking process used affects the physical properties of the resulting hydrogel (mechanical strength, heat resistance, and solvent resistance).^8^

**Table 1:** Advantages of Reactions and Methods Involved in Some Chemical and Physical Crosslinking methods^8^

Method of cross-linking	Reactions or interactions involved	Advantages
Chemical	Photochemical reactions^a^	Performed at physiological temperature and *p*H. Noninvasive and nontoxic.
	Enzymatic reactions	Limits release of potential toxic byproducts of side reactions. Highly selective and cell compatible.
	Click reactions^a^	High rate and efficiency, favorable reaction conditions and biologically compatible.
Physical	Ionic cross-linking	High toughness.
	Temperature dependent methods	Do not require chemical stimulants, highly biocompatible and high sensitivity to body temperature.
	*p*H dependent methods	Able to stimulate and respond to environmental changes.

a Note that not all abiotic photochemical reactions and click reactions are biocompatible.

## Hydrogel Properties

Important properties of hydrogels to be considered are the swelling ratio and water absorption of the hydrogel, their mechanical properties, and their biological properties.^8^ The degree of hydrogel swelling is directly linked to the degree of cross-linking.^15^ Mechanical properties include but are not limited to elasticity and strength, whereas thermal properties include phase transition temperature among other properties. The rheological behavior of hydrogels, i.e., their flow and deformation characteristics, is of importance when designing a hydrogel for a specific application. These characteristics determine how the hydrogel can be administered, stored, and used. All of these properties must be determined during the characterization of hydrogels. The characterization of these properties has been extensively reviewed.^16^

## Biodegradability and Reversibility of Hydrogels

For the biodegradability of hydrogels, labile bonds are incorporated into either the network backbone or in the cross-links.^17^ These bonds can be cleaved under physiological conditions using different procedures including solubilization, chemical hydrolysis, enzymatic hydrolysis, ionization or ion exchange, and photochemical deprotection.^17–19^ There has been a movement from static hydrogel systems that follow simple degradation to dynamic hydrogel systems that respond to internal biological or external signals with spatial precision.^20^ Hydrogels of particular interest for wound management, for example, are those that can cross-link in situ and dissolve on-demand using physical or chemical reactions.^21^ In general, hydrogels for biological applications must mimic the viscosity and stiffness of the surrounding tissues and allow for the selective permeation of essential cells and nutrients. This is required to prevent negative cellular responses to abnormal mechanical signaling.^22^

## Hydrogels as Contraceptives

The use of hydrogel technology in contraception is a relatively new area of research with great potential. In a study conducted by Hardy et al., a total of 635 women (including adolescents and adults from low and middle-high socioeconomic groups) were interviewed on their preferences for different dosage forms (gels, creams, tablets, foams, films, and suppositories) of contraceptive formulations. The results of this study indicated that the majority of women (40% of the candidates) preferred gels over other vaginal formulations.^23^ The way in which a formulation provides contraception may influence the acceptance (individually or culturally) of a contraceptive method.

Understanding the mechanism of action (MOA) of contraceptive methods is important when developing new methods or improving existing formulations. The MOA behind hydrogels as contraceptives has not been discussed in depth. There are several different MOAs behind hydrogel contraceptives, each with its advantages and disadvantages, that are being explored (Table 2). The most common of these MOAs include physical barriers, spermicidal effects, controlled drug release, and combination approaches (Figure 3). There are several other less common MOAs being investigated, including immunological approaches, sperm immobilization, cervical barrier enhancement, and targeted delivery. The specific MOA for a particular hydrogel-based contraceptive depends on its composition and design and most of these contraceptives are either in early stages of development, or still in pre-clinical or early clinical trials. Ongoing research to optimize the properties of these hydrogels to improve on biocompatibility, biodegradability, and controlled release duration, as well as further evaluations of their safety and efficacy is, in most cases, still required.

**Table 2:** Some Advantages and Disadvantages of Mechanisms of Action for Hydrogels Designed for Contraceptive Use

Mechanism of Action	Advantages	Disadvantages
Physical Barrier	Simple	May be irreversible
	Effective	May lead to epididymis damage
	Longer lasting	May cause vas deferens or vaginal/cervical tissue damage
	Nonhormonal	May lead to pain or discomfort
	Not harmful to sperm	
	Use in male and female contraception	
Spermicidal Effects	Nonhormonal	Low efficacy
	Use in male and female contraception	Harmful to sperm
		Typically requires additional tests for regulatory approval
Controlled Drug Release	Either hormonal or nonhormonal	More complex than other MOAs
	Use in male and female contraception	Short-term contraception
	Multiple mechanisms for contraception	Hormonal options may have unwanted side effects
	Increases efficacy of drugs or hormones used	Typically requires additional tests for regulatory approval
	Reduces systemic side effects of contraceptive drugs	Requires extensive additional research on drugs encapsulated in hydrogels
Combination Approaches	Potential efficacy enhancement	More complex than other MOAs
	Use in male and female contraception	
Immunological Approaches	Novel	Not well studied
	Nonhormonal	May be irreversible
	Use in male and female contraception	Complicated design
		Efficacy and viability not known
		Not used in humans yet due to unknown side effects
Sperm Immobilization	Nonhormonal	Not well studied
	Use in male and female contraception	May be harmful to sperm
		Short-term contraception
		May need to be coupled with other MOAs
Cervical Barrier Enhancement	Longer lasting	May be irreversible
	Nonhormonal	Female contraception only
		May lead to cervical barrier damage
Targeted Delivery	Either hormonal or nonhormonal	More complex than other MOAs
	Use in male and female contraception	Short-term contraception
	Multiple mechanisms for contraception	Hormonal options may have unwanted side effects
	Increases efficacy of drugs or hormones used	Requires extensive additional research on drugs encapsulated in hydrogels
	Reduces systemic side effects of contraceptive drugs	

**Figure 3:**
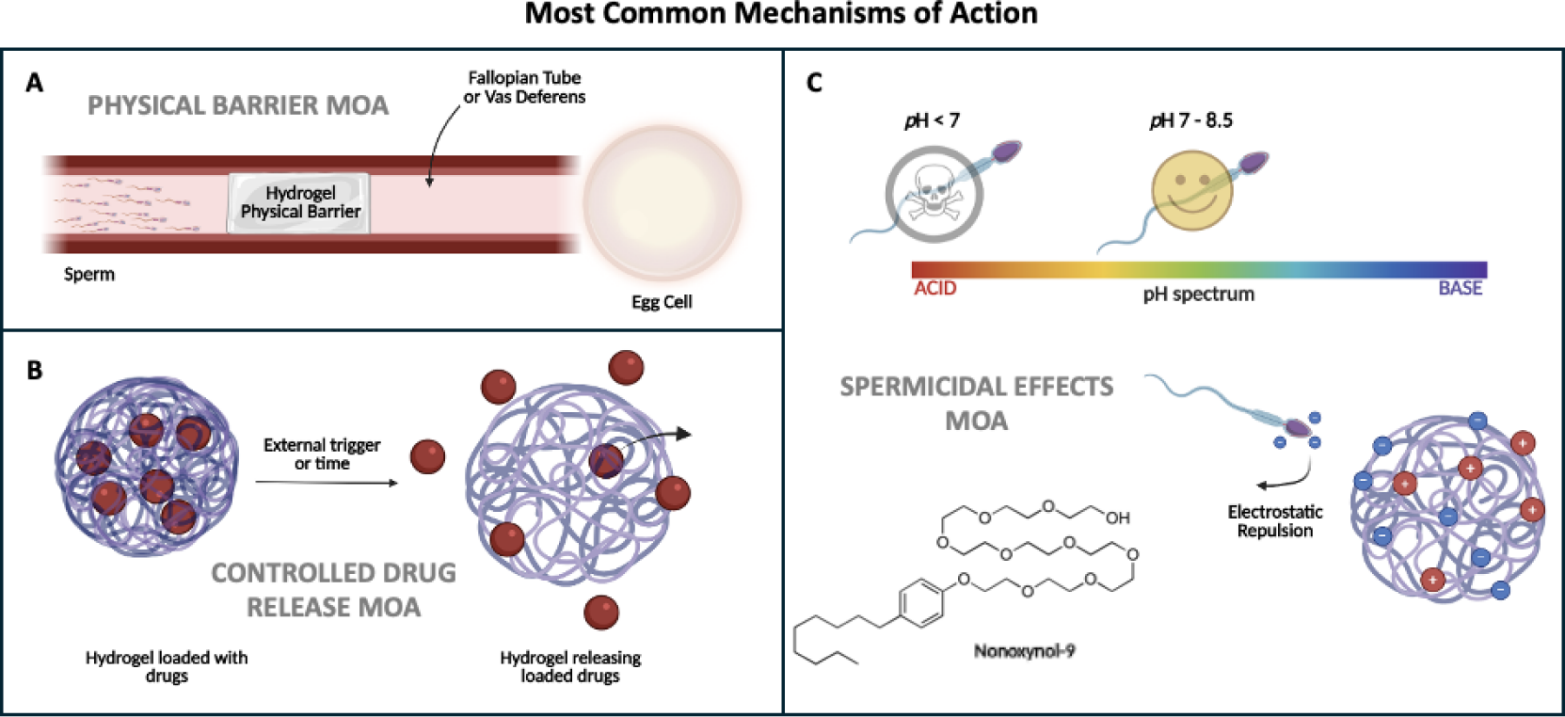
Most common mechanisms of action. A: Physical barrier MOA – prevention of sperm from reaching an egg cell; B: Controlled drug release MOA – drug-loaded hydrogel releasing drugs over time or due to an external trigger; C: Spermicidal effects MOA – acidic environment leads to loss of sperm function, nonoxynol-9, a common spermicide used in contraceptives, and repulsive electrostatic forces on hydrogel affecting sperm mobility (Images created using BioRender).

## Physical Barrier

In the physical barrier MOA, hydrogels can be used as occlusive devices, whereby they act as physical barriers somewhere within the reproductive tract, inhibiting the movement of sperm (Figure 3).^22^ Hydrogels that can form in situ can solidify within the vagina (typically the fallopian tubes), or within the vas deferens, creating a physical barrier and blocking the passage of sperm (Figure 4). Increased cross-linking density leads to stiffer gels with smaller pore sizes and, hence, decreased particle mobility.^22^ The method by which the hydrogel is formed, the polymer concentration within the gel, as well as the hydrogels’ responses to stimuli, may affect its pore size and must be taken into account when designing a hydrogel as an occlusive device.^22^ The physical barrier mechanism is ideal for designing simple hydrogels that could be used by both men and women, as they do not include the use of hormones or drugs that are specific to each sex.

**Figure 4:**
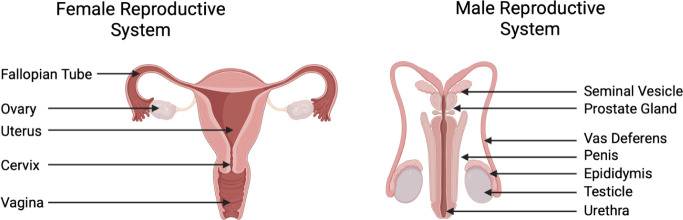
Human reproductive systems (left, female; right, male) (image created using BioRender).

In the early 1980s, the first occlusive hydrogel device for this application was studied. RISUG (Reversible Inhibition of Sperm Under Guidance) is a “non-occlusive” styrene maleic anhydride product that lines the vas deferens, using an apparent change in *p*H to create a “positively charged” complex that disrupts the sperms’ acrosomal layer, preventing fertilization for up to 10 – 15 years.^24–28^ This intravas injection technology was studied in nonhuman primates and was found to effectively prevent fertilization and could be successfully reversed with a vibratory/percussive massage.^28^

Guha suggested that the proposed MOA involves the formation of a positively charged SMA-DMSO-amino acid complex that leads to the disruption of sperm surface enzymes and lipid domains, increasing the membrane fluidity and, in turn, destabilizing the sperm membranes, essentially causing a loss of fertility. It was proposed that the low *p*H conditions caused by the complex, in combination with the complex electrical charges of the polyelectrolyte system, may produce sperm abnormalities and hence affect fertility.^29^ These effects were hypothesized to be enhanced in the presence of proteins from bodily fluids.^30^ This hypothesized MOA has not yet been confirmed. The contraceptive MOA is more likely that of a physical barrier, whereby the hydrogel is used to block the passage of sperm.

The reversibility involving either the injection of a DMSO or sodium bicarbonate solution, or percutaneous squeezing of the vas deferens, along with electrical stimulation and vigorous massaging of the hydrogel, has been successfully shown in numerous animal studies but has not yet been studied in humans.^28^ This product has been studied over several decades and has undergone multiple animal studies (rats, rabbits, and monkeys) and human clinical trials; however, it has never been brought to market.^31^

The P-block, Mark 9, was developed in the 1980s (around the same time as RISUG developments started) by Brundin et al. as an occlusive intrauterine contraceptive.^32,33^ It was described as a “hydrogelic intratubal device” that was inserted hysteroscopically into the fallopian tubes, whereby the dried hydrogel swelled in the presence of bodily fluid to prevent intratubal and intrauterine pregnancies. This formulation is different from the RISUG formulation, whereby it is inserted as a preformed (dried) gel, as opposed to an in situ-forming hydrogel. The device consisted of a nylon-6 core on which a hydrogelic body was attached by copolymerization using γ-irradiation. The P-blocks were successfully inserted into 22 women, whereby none of them became pregnant for up to 6 months after insertion. The P-block, Mark 9, was designed as an occlusive device; however, the exact nature of the antifertility action is still unknown. It was designed for permanent contraception; however, after removal, successful pregnancy was observed.

In 1994, Maubon et al. developed and tested an occlusive hydrogel implant consisting of a copolymer of acrylonitrile dissolved in DMSO and saline, certain dyes, and a sclerosing agent (for tubal wall alteration) in rabbits.^34^ The intrauterine device showed promise for contraception in female rabbits; however, it was found to have negative side effects such as tubal inflammation, and the hydrogel was not mechanically strong enough, whereby it fragmented in the tube and did not remain intact after insertion. Since the gel is mechanically weak, the tubal inflammation is likely due to the sclerosing agent, indicating that tubal wall alteration may not be the most ideal way to cause occlusion in the uterus or fallopian tubes.

Ushercell, a uniquely high molecular weight form of sodium cellulose sulfate, was developed in 2002 and evaluated in phase 1 clinical trials as a contraceptive antimicrobial agent by Anderson et al.^35^ It is active against both spermatozoa and multiple sexually transmitted infections (STIs) including HIV, chlamydia, and herpes, and has been shown to be nontoxic and to have an acceptable safety profile via preclinical toxicity studies.^36^ This patented product was found to be an effective contraceptive when vaginally applied, and the mechanism of contraception is still unknown. It was hypothesized to have several effects on sperm function, ultimately immobilizing sperm, as well as acting as a barrier, preventing the penetration of sperm in cervical mucus; however, additional studies are required to confirm the possible MOA of contraception of Ushercell. The additional antimicrobial properties of the hydrogel make it more desirable as a contraceptive.

It was shown to prevent conception in rabbits when added to sperm, as well as when applied to the vagina prior to insemination.^36^ Further studies of Ushercell indicated that it is a reversible, effective, long-lasting (up to 24 h) contraceptive at relatively low concentrations (6% cellulose sulfate gel).^36^ Furthermore, the safety and efficacy of the 6% cellulose sulfate gel were studied in humans, and it was found to be safe for use twice daily over a two-week period.^37^ Phase 3 clinical trials of Ushercell as an HIV microbicide were stopped when it was found to increase the rate of HIV infection in the women enrolled in the randomized trial.^38^ The increased rate of HIV infection in women who used Ushercell indicates the importance of hydrogels undergoing various tests (including those related to STIs) before being commercialized, to identify the increased risks associated with using hydrogels for contraceptive purposes. This would include hydrogels that are not designed for STI prevention or treatment, as the presence of the hydrogel or the damaging effects it may have on the surrounding tissue may lead to an increased risk for infection.

Vasalgel, another intravas insertion product, was developed in 2011 by the Parsemus Foundation as a long-term, reversible contraceptive and is currently under further development as Plan A by NEXT Life Sciences, a US-based biotechnology company. This technology uses a high molecular weight SMA acid polymer that is dissolved in DMSO, and upon insertion into the vas deferens, swells to fill the lumen, and acts as an occlusive device, preventing the passage of sperm upon ejaculation.^39^ The formation of Vasalgel relies on nonsolvent-induced phase inversion, whereby, upon insertion into the body, the DMSO is rapidly exchanged with bodily fluid, transforming the polymer solution into a physically cross-linked hydrogel. Vasalgel has been shown to reliably prevent the passage of sperm in different animal studies and was proven to be reversible after over 1 year using a sodium bicarbonate solution in a rabbit model.^40,41^ This product is expected to enter clinical trials in the near future and shows promise for commercialization. This formulation was based on the original RISUG formulation.

In 2019, the RISUG formulation of SMA was investigated as a nonhormonal fallopian tube implant for female contraception.^42^ The hydrogel exhibited selective antimicrobial activity as well as excellent biocompatibility in rats, and it was concluded that the formulation showed great promise for the development of an intrauterine implant for female contraception. Furthermore, it was hypothesized that RISUG may impart potential anticancer properties and could, in addition to providing contraception, assist in the prevention of early-stage endometrial cancer post-implantation.^43^ This hypothesis has not yet been validated by in vitro or in vivo studies. This formulation would be ideal if it could be used for both male and female contraception.

In 2020, Subramanian et al. investigated a flexible block copolymeric scaffold of RISUG, blend-grafted with poly­(ethylene glycol)-modified polycaprolactone, for use as a biodegradable, nonhormonal, female intrauterine long-term contraceptive device.^44^ In vitro studies showed that the spermicidal activity of the grafted polymeric scaffold increased as the concentration of SMA hydrogel within the scaffold increased. The blend was shown to be biocompatible in organ pathology tests and nontoxic to rat uterine cell lines. Further investigations to determine the long-term efficacy of contraception are yet to be done, as well as in vivo contraceptive studies.

Wurm et al. investigated the use of κ-carrageenan physically cross-linked hydrogels for the reversible occlusion of the vas deferens.^45^ This formulation is different from most of the other hydrogel systems for contraceptive purposes, as it makes use of naturally derived polymers as opposed to synthetic polymers, which could lead to increased biocompatibility. After extensive swelling studies, it was found that the gels were feasible candidates for occlusion of the vas deferens.^46^ They used a gel-tube model to test whether the hydrogel could be removed by using compressive disintegration. They found that manually applied pinch forces were not enough to completely disintegrate the gel matrices for reversal and removal of the gel in the modeled vessels. It was suggested that compressive devices might assist in the disintegration of the hydrogels but may result in undesired damage to surrounding tissue. Further biocompatibility and toxicity studies are yet to be completed for this formulation. This study focused on the very important aspect of ease of reversal/removal, which many other studies did not focus on. Chemical reversal would likely cause less damage than physical reversal and should be considered for future formulations.

Adam is a more recent hydrogel technology developed around 2022 by a US-based biotechnology company, Contraline. This hydrogel acts as an occlusive device within the vas deferens and is based on a two-component aqueous system that, once mixed, forms a hydrogel. This product has already entered human clinical trials, whereby, after the injection of the technology into 23 men in Australia, the sperm count was reduced by 99–100%. This contraceptive has been tested in an animal study and was shown to last up to 2 years, with the option of being reversed at an earlier stage. The reversal has, however, not yet been tested in humans. The chemistry behind this technology is based on a PEG polymer system cross-linked by thiol-maleimide click reactions.^47,48^

An alternative two-component aqueous system based on modified SMA and a PEG-based cross-linker was developed by Klumperman and coworkers.^49^ The chemically cross-linked hydrogel contains thioesters within its cross-links, which can be reversed through native chemical ligation to redissolve the hydrogel on demand.^50^ This patented technology is still in its early developmental stages. Wang et al. developed an ultrasound-induced self-clearance hydrogel composed of sodium alginate conjugated with reactive oxygen species-cleavable thioketal, titanium dioxide, and calcium chloride.^51^ TiO_2_ was used as a sonosensitizer to generate the reactive oxygen species after ultrasonification, and CaCl_2_ was used to trigger the hydrogel formation. A solution of these materials was injected into the vas deferens and formed a hydrogel in situ within 160 s. The reversal relied on the use of remedial ultrasound, which triggered the production of reactive oxygen species by titanium dioxide, which inherently cleaved the sodium alginate-thioketal conjugate, resulting in the dissolution of the hydrogel. The insertion and reversal of the hydrogel could be monitored by using diagnostic ultrasound. This technology was evaluated in vivo using pubescent SD rats, and effective contraception was achieved. Fertility after reversal using remedial ultrasound was completely restored.^51^ This formulation includes commonly available methods for visualizing implantation and reversal within its design, which adds a level of superiority over other formulations that do not include methods for visualization. This is an important consideration, as confirmation of insertion (and removal) of a contraceptive is imperative for ensuring successful prevention of conception. The complexity of the formulation could possibly lead to increased costs in production as well as an increased risk of failure of gelation or reversal.

Anthis et al. developed a stimuli-responsive hydrogel as a reversible mechanical female contraceptive that could also be used for endometriosis treatment.^52^ The hydrogel was designed to reversibly occlude the fallopian tubes and was comprised of two different acrylamide-based polymers cross-linked with a disulfide cross-linker (*N,N*’-bis­(acryloyl)­cystamine) or a photolabile poly­(ethylene glycol) diphotodegradable acrylate cross-linker, whereby noninvasive reversal (within 30 min) used disulfide-reducing agents and near-visible UV light, respectively. The hydrogels were inserted as dried hydrogel materials, which swell in the presence of bodily fluid to fill a portion of the fallopian tube, ultimately preventing the passage of sperm, as well as endometrial cells. The hydrogels were found to be biocompatible and noncytotoxic when tested in fresh porcine fallopian tubes and could easily be inserted using readily available gynecological tools. Insertion could be visualized using ultrasound guidance. Further in vivo human clinical studies are required to confirm the compatibility and functionality of the gel, as well as to determine the integrity of the fallopian tube after reversal. Furthermore, post-removal fertility must also be investigated. This formulation also includes methods for visualization during insertion, which makes it easier to confirm successful contraception. These last two formulations are very quick to insert, as well as to remove, making the procedures involved during insertion and removal more cost-effective and less invasive. The added visualization aspects make them both more appealing than the other formulations.

## Spermicidal Effects

Hydrogels that are acidic in nature can be used to lower the *p*H of the vagina, creating a hostile environment for sperm, leading to reduced motility or immobilization and reduced viability.^53^ Repulsive electrostatic forces can also inhibit particle mobility in hydrogels, further increasing their ability to block the flow of charged particles.^22^ Alternatively, the hydrogels can be loaded with spermicides/sperm-killing agents, allowing for sustained release and localized action, leading to sperm destruction (Figure 3). Many of the gels with spermicidal properties need to be used in conjunction with an additional contraceptive due to the low efficacy of contraception on their own. Contraceptives that have spermicidal and/or hormonal effects require additional tests for FDA approval and are, therefore, more costly to study for use inside the human body.

In 1982, Singh et al. investigated the use of a poly­(2-hydroxyethyl methacrylate-*co*-methacrylic acid), poly­(HEMA-MAA), hydrogel for use as a male contraceptive.^54^ The polymer solution consisting of poly­(HEMA-MAA) dissolved in DMSO is inserted into the vas deferens, whereby the DMSO and water are exchanged, resulting in the formation of a hydrogel. Spermicidal action in vitro studies indicated that sperm became immediately immotile when in contact with the hydrogel, and it was concluded that the spermicidal action was due to the low *p*H environment caused by the presence of the carboxylic acid moieties from the methacrylic acid repeat units. In vivo fertility action was tested using rats, and the results indicated that, in the presence of the hydrogel, no fertility was observed. The presence of dead, whole, or decapitated sperm in vaginal smears post-coitus indicated that the hydrogel did not act as a barrier but rather with spermicidal effects to hinder fertility. The intravasal gel was able to be flushed out with DMSO.

BufferGel was developed by ReProtect, LLC, as an aqueous vaginal gel with a *p*H of 3.9, equipped with an acidic buffering action designed to hinder vaginal neutralization by semen and, hence, act with an irreversible spermicidal effect.^53,55^ This high-molecular-weight, cross-linked, poly­(acrylic acid) gel has the ability to buffer twice its volume of semen to a *p*H lower than 5 due to its active ingredient, the hydrogen ion. Not only is BufferGel spermicidal, but it is also virucidal to HIV and herpes simplex virus type 2, among other STIs. This gel was shown to have a low toxicity profile, and minimal side effects were observed by those who underwent clinical trials (low-risk, abstinent women and monogamous women). The most common adverse side effect was irritative genitourinary symptoms.^55^ Further evaluation of the contraceptive efficacy of BufferGel in humans is still required, including postcoital tests and contraceptive trials; however, the contraceptive efficacy of BufferGel with a cervical barrier (such as a diaphragm) has been tested.^56^ Furthermore, the BufferGel Duet, a buffering microbicide and spermicide gel applied to the cervix via a novel applicator, was developed and tested, and the study indicated successful use and application of the applicator, showing feasibility for further development of this technology.^57^

## Controlled Drug Release

The spermicidal MOA is related to controlled drug release. The controlled drug release MOA can be either hormonal or nonhormonal. In hormonal controlled drug release, hydrogels can encapsulate hormones like progesterone, which inhibit ovulation or thickening of cervical mucus, and slowly release these hormones, leading to decreased chances of fertilization (Figure 3). Nonhormonal options can include other compounds with contraceptive effects, such as antiprogestins, which prevent implantation, or vasodilators, which cause blockages in the vas deferens.

In 1993, Shantha et al. designed a biodegradable hydrogel based on poly­(ethylene glycol) (conjugated with/forming a physical adduct with collagen) and poly­(*N*-vinylpyrrolidone), externally cross-linked using hexamethylene diisocyanate to be used as a reversible, hormonal male contraceptive.^58^ The hydrogel was loaded with testosterone, a male contraceptive steroid, and showed a zero-order release profile after an initial burst release for up to 90 days, after which the study was discontinued. The downfall to this formulation could be the incorporation of high levels of testosterone, as increased testosterone levels in men may lead to adverse side effects and an increased risk of complications.

D’Cruz et al. describe gel-microemulsions that could be used as intravaginal/rectal delivery vehicles for pharmaceutical drugs with activity against STIs, as well as those that exhibit spermicidal activity in human semen.^59^ The contraceptive efficacy of the gel-microemulsions was proven in rabbit model studies, and intravaginal toxicity was tested in rabbits and mice, where the formulations were found to be safe and nontoxic. These gel-microemulsions are to be used continuously and are not “permanent but reversible”, which leads to similar issues that are currently faced with the use of condoms such as failure to use them correctly, if used at all.

The use of gels in vaginal drug delivery systems was reviewed in 2006 by das Neves et al., whereby the use of vaginal gels as contraceptives was briefly discussed.^60^ A few examples of gels loaded with spermicidal drugs or contraceptive agents and gels with acid-buffering capabilities were mentioned, including some marketed vaginal contraceptive gels such as Advantage-S, Conceptrol, and Gynol (II) (all loaded with nonoxynol-9 as the contraceptive agent). Again, the use of gels as a delivery system for contraceptive drugs is typically designed to be temporarily used prior to or immediately after intercourse, which may lead to failed contraception due to incorrect use/application.

Nonoxynol-9 (*N*-9) has been used over the past 60 years globally as a spermicide for the killing of sperm for contraceptive purposes (Figure 3). It has been shown to have multiple negative side effects but is the active ingredient in multiple commercially available contraceptives. It has been loaded into gels and, as such, has been used as a vaginal contraceptive. The effect of *N*-9 on sperm functions was systematically reviewed by Xu et al., who summarized details of the different delivery systems, including those of gels.^61^

Jalalvandi et al. developed a *p*H-responsive hydrogel based on the mucoadhesive biopolymer, chitosan, for anticancer and contraceptive purposes.^62^ Fast- and slow-degrading hydrogels were developed (tuned using cross-linking density) and were loaded with a nonhormonal spermicide and an anticancer agent, namely iron­(II) gluconate dihydride and doxorubicin hydrochloride, respectively. The hydrogel is formed after insertion and degrades over time to release the therapeutic agents intravaginally. For nonhormonal contraceptive purposes, the hydrogel is to be inserted inside the vagina prior to intercourse, whereby the spermicidal agent is quickly released. The cytotoxicity of the hydrogels was tested against mesenchymal cell lines and showed no cytotoxic effects. Further in vivo studies are still required for in-depth cytotoxicity studies and mucoadhesive assessment.

Long et al. developed a controlled-release system for levonorgestrel, using chemically cross-linked chitosan microspheres embedded in a physically cross-linked poly­(vinyl alcohol) hydrogel for long-term contraceptive delivery.^63^ A zero-order release profile (without burst release) was obtained for the microsphere-hydrogel systems, and this system was considered to be promising as a long-term contraceptive delivery system. This type of once-off insertion with long-term contraceptive delivery would be ideal over insertion prior to intercourse for short-term/immediate delivery.

Xie et al. developed a promising carbomer-based trifunctional contraceptive hydrogel for intravaginal administration.^64^ This contraceptive gel was loaded with three FDA-approved drugs, namely tenofovir, gossypol, and nitroglycerin, which each play a role in the prevention of STIs, contraception, and male erectile function, respectively. Gossypol was confirmed to inhibit sperm motility of pig sperm samples and is hence the spermicidal agent released by the hydrogel. This gel was tested for its safety and functionality using multiple in vitro experiments and was shown to successfully prevent conception in female rats when intravaginally applied and enhance erectile function in rats when applied to male genitalia. The inhibitory effect on STIs was not verified using animal models. This formulation is used prior to intercourse; however, it has the added benefit of enhancing erectile function, which may be appealing to men with erectile dysfunction or associated disabilities.

Recently, much research has gone into the use of microneedles for the controlled release of therapeutics.^65^ Microneedle patches have been developed for the slow controlled release of hormones for female contraceptive use.^65–68^ A particularly interesting type of microneedle is that of hydrogel-forming microneedles.^66^ These have not yet been explored for contraceptive use but should be considered during the design of new hydrogel-based contraceptives.

Wang et al. synthesized a contraceptive drug-loaded composite hydrogel composed of modified cellulose (aldehyde-replaced oxidized regenerated cellulose) and chitosan, cross-linked via a Schiff base reaction.^69^ The contraceptive drug, a drospirenone liposome, is poorly water-soluble; hence, its incorporation into a hydrogel allows for the controlled slow release of the drug and increased absorption and efficacy of the contraceptive, reducing side effects associated with increased oral intake. The promising results indicated that the hydrogel was successfully synthesized and the drug was successfully loaded into the hydrogel, leading to improved drug solubility and stability.

## Combination Approaches

Combination approaches combine two or more mechanisms within one hydrogel formulation, potentially enhancing the efficacy of the hydrogels and providing increased prevention of unwanted pregnancy. Some of the above-mentioned formulations could also be considered for the combination approach MOA; however, they were classified according to their suspected primary cause of contraception.

A cocktail-inspired male contraceptive was developed by Bao et al., and it relies on both chemical and physical mechanisms for effective contraception.^70^ The hydrogel formulation is a mixture of sodium alginate, calcium carbonate, and gluconolactone, which make up the injectable hydrogel. EDTA is the agent used to provide chemical contraception, as it has the ability to inhibit sperm motility; however, it is also used to dissolve the hydrogel upon reversal. The contraceptive formulation is inserted into the vas deferens by the sequential injection of PEG-Au nanoparticles (used as a temperature-switchable physical barrier to encapsulate the EDTA), followed by EDTA (for sperm inhibition and hydrogel dissolution), another layer of PEG-Au nanoparticles, and finally the calcium alginate hydrogel (used as a long-term physical barrier). Near-infrared irradiation is used for the reversal of the hydrogel, whereby after 5 min of irradiation, the PEG-Au nanoparticles melt, allowing the EDTA to gradually dissolve the hydrogel, restoring fertility. This contraceptive method was shown to prevent conception in rats for more than 2 months, indicating that it is an effective medium-term contraceptive. Further experiments are required to determine the safety of the materials as well as to determine the exact duration of contraception. The complexity of this formulation may lead to increased variables for potential failure.

## Immunological Approaches

The less common approaches require further studies to confirm the viability and efficacy. For example, immunological approaches can include hydrogels loaded with third-party modulators, such as antigens, that can induce an immune response that specifically targets sperm cells, leading to infertility.^22^ This approach can face challenges in antigen design and safety. The idea of antispermatozoal antibodies was investigated by Jager and Kremer et al.^71–74^ Immunocontraception is not currently approved for human use; however, researchers have been studying the use of hydrogels as delivery vehicles for immunocontraceptives in animals.

For example, Bansal et al. developed an adjuvanted hydrogel-based *p*DNA nanoparticulate vaccine for immunocontraception and rabies protection and tested it in mice.^75^ The results indicated that after the mice were exposed to the hydrogel and nanoparticles, anti-GnRH antibodies (which cause the immunocontraceptive effect) were present for up to 12 weeks. Further studies on the efficacy of the nanoparticles in causing sterility in the animals are still required.

Wu et al. developed a thermoresponsive chitosan hydrogel for use as an antirabies and immunocontraceptive vaccination.^76^ Initial studies on the ERA-2GnRH vaccine alone indicated that it successfully provided protection from the rabies virus and led to >80% infertility in mice after triplicate doses. The vaccine was then loaded into a hydrogel for safer sustained release of a one-dose delivery of the formulation, whereby the antirabies properties were improved, but the contraceptive properties of the vaccination were compromised. It was suspected that due to the slow release of the gonadotropin-releasing hormone antibodies, an upper threshold required for contraception was not achieved. Further improvements on the formulation and delivery methods are required. This formulation is superior to that developed by Bansal et al. as it includes antirabies properties.

Since this MOA is not yet accepted for use in humans, it will likely only be used in formulations that target animal sterilization. The effects of altering immune responses need to be studied in more depth before this type of technology is commercialized for widespread use in animals and/or humans.

## Sperm Immobilization

Hydrogels can be designed to have adhesive properties, leading to the capture and immobilization of sperm within the reproductive tract, or they could be loaded with chemoattractants, whereby, in both cases, movement toward the egg is prevented.

Patel et al. designed a curcumin-loaded in situ forming hydrogel for female contraception utilizing a Box-Behnken statistical design for optimization.^77^ Poloxamers are copolymers composed of poly­(ethylene oxide) and poly­(propylene oxide) units, which have the ability to form gels near body temperature. Poloxamer 407 (P407) is an ideal filler used in the preparation of temperature-sensitive, in situ forming gels, as it has good temperature sensitivity and biocompatibility, whereas poloxamer 188 (P188) is used to regulate the gelation temperature. Different formulations of mixtures of P407/188 and a mucoadhesive polymer hydroxypropyl methylcellulose (HPMC K4M) were compared and optimized, and the final formulation was loaded with curcumin. When subjected to sperm immobilization studies, the formulation was shown to successfully immobilize sperm within 11 s. The hydrogel was designed to be placed within the vagina, 30 min prior to intercourse, to allow sufficient time to release 50% of the drug to achieve contraceptive efficacy. It was hypothesized that both curcumin and poloxamer contributed to sperm immobilization. Preclinical studies are still required prior to testing in women. In addition to in vitro contraception, the dosage form could also be placed inside a condom for additional spermicidal action. Again, the hydrogel is intended to be used prior to intercourse, allowing for potential risks of failed contraception due to a failed or forgotten application.

Not many hydrogel formulations have been developed to target this type of mechanism of action, possibly because this mechanism of action is not effective on its own and may need to be coupled with additional modes of action to ensure effective contraception. Further research is required to confirm this claim.

## Cervical Barrier Enhancement

Hydrogels can be used to thicken or stiffen cervical mucus, creating another type of physical barrier to sperm passage. This MOA is similar to existing cervical cap methods but has the potential for longer-lasting and/or more comfortable use. Saxena et al. describe a biodegradable hydrogel composed of dextran, copolymers of polylactide, and ε-caprolactone for use as a nonhormonal, intravaginal contraceptive device.^78^ The hydrogel was loaded with different spermiostatic drugs in order to have multiple uses and effects. Iron­(II) α-gluconate dihydrate was used for the immobilization of the sperm tail due to lipid peroxidation; ascorbic acid was used to thicken the cervical mucus to prevent sperm penetration; mixtures of polyamino and polycarboxylic acids were used to maintain a vaginal *p*H of approximately 4.5. The combination of these drugs ensured that sperm was not motile and could not survive the vaginal environment. The hydrogel was shown to elute effective combinations of these drugs within 30 s for up to 16 days using sperm penetration tests, and in vivo studies were carried out on rabbits indicating successful killing of sperm after insemination. This is the only hydrogel formulation that was designed to enhance the cervical barrier to prevent the passage of sperm; however, it could also be classified under the combination approaches due to the additional sperm immobilization properties.

## Targeted Delivery

Targeted delivery MOA can potentially reduce systemic side effects of contraceptive drugs by allowing these drugs to be delivered directly to their target sites. This can be achieved by designing the hydrogel to adhere to specific cells or tissues or by designing a hydrogel that is responsive to physiological changes, the latter allowing the release of the contraceptive drug (e.g., progesterone or levonorgestrel) only in response to specific conditions such as hormonal fluctuations or the presence of sperm, indicated by changes in *p*H or temperature, for example. Most examples of this kind found in the literature suggest contraceptive applications; however, the hydrogels in question were not tested for specific contraceptive applications.^79,80^

## Challenges and Future Directions

Many different hydrogels as contraceptives have been designed and tested over the past couple of decades. The main issue that most of these formulations have faced is biocompatibility and increased side effects. Many of the designed hydrogels caused discomfort or irritation in users. A comfortable, completely biocompatible option has yet to be commercialized. One big hurdle for all researchers in this field is the requirement for essential preclinical and clinical trials, which are expensive and time-consuming. Many of the options that have reached clinical trials were abandoned due to the toxicity or negative effects experienced by the patients involved. The safety, efficacy, and regulatory hurdles stand in the way of researchers; however, these parameters and hurdles are very important and cannot be avoided in the design of contraceptives, especially those that end up inside the human body.

It is clear from Figure 5 that the majority (71%) of the hydrogels designed for contraceptive use targeted female contraception, and the physical barrier and controlled drug release mechanisms of action were the most common for both men and women. There is a clear gap in the research targeting hydrogels as male contraceptives, and many of the less common MOAs have little to no hydrogel designs published in the literature.

**Figure 5:**
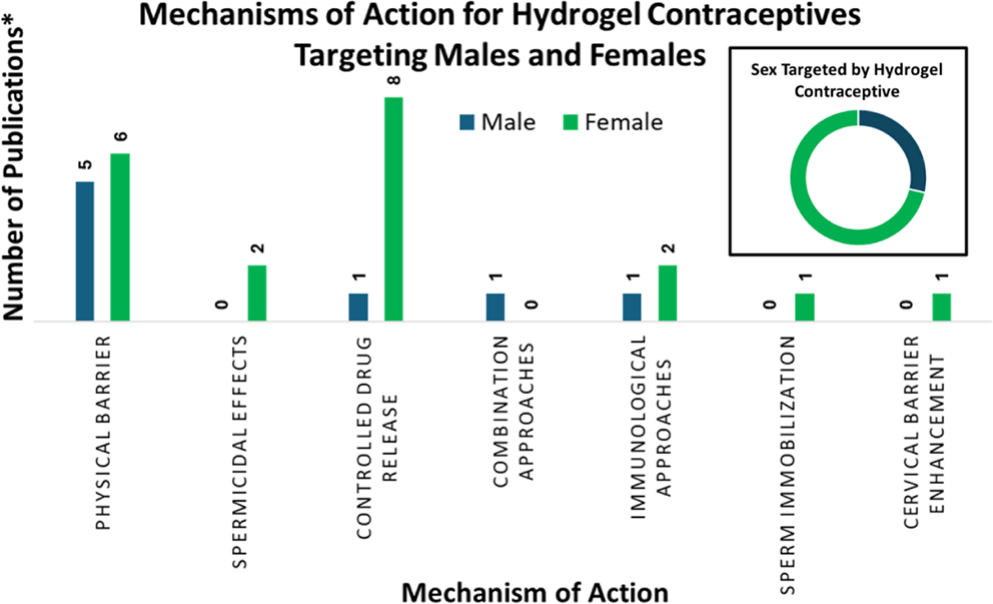
Comparison of mechanisms of action for hydrogel contraceptives for different sexes *Publications were sourced from the PubMed NIH database, using keywords “hydrogel contraception” searching the literature published between 1973 and 2024. Furthermore, the Scopus database, using keywords “hydrogel AND contraceptive OR contraception” searching literature published on any date was also used. Additionally, reference lists of relevant papers were used as an additional source of papers.

Increased research in this field will likely lead to an accepted and widely used nonhormonal contraceptive, for both men and women, that avoids the complications involved with hormonal contraceptives. An occlusive, nonhormonal medical device that is suitable for both men and women would be an ideal output from this growing field of research.

## Conclusion and Summary

Although much research has been conducted on the use of hydrogels as contraceptives, the majority of the research has targeted female contraception (Figure 5). In addition to this, most of the hydrogels were not designed to be used as a “permanent-but-reversible” contraceptive but rather designed to be applied prior to intercourse.

From the research that has been conducted, it is clear that hydrogels have great potential to act in a contraceptive manner and can also be designed to include additional microbicidal and antiviral properties, adding to the advantages of using this type of technology as a contraceptive. Understanding the mechanisms of action of hydrogels as contraceptives is important for further optimizations and future designs. Hydrogels as contraceptives form a promising, understudied research avenue with great potential for both male and female contraception. Research into the use of hydrogels as contraceptives started decades ago, and recent developments of nonhormonal hydrogels as contraceptives are promising alternatives for both men and women due to the decreased side effects when compared to currently available hormonal options. In particular, these kinds of nonhormonal options are more appealing to men, as men are not as interested in hormonal options as women are.

The development of a long-lasting, nonhormonal, completely reversible contraceptive for both men and women would be the most ideal contraceptive option. In particular, for men, an option of this kind would allow men to contribute to family planning decisions and the prevention of unwanted pregnancies, leveling out the gender inequalities that men currently face in terms of contraceptive options and availability. More nonhormonal, reversible contraceptive options in general would allow women to have better reproductive health. Effective family planning for both men and women may lead to a reduction in abortion rates and could enhance maternal and newborn health. Despite the limited publications in this growing field, a new hydrogel contraceptive is likely to be commercialized in the near future.

## Search Strategy

Examples from the literature on hydrogel-based contraceptives were sourced from the PubMed NIH database using keywords “hydrogel contraception” searching for the literature published between 1973 and 2024. Furthermore, the Scopus database using keywords “hydrogel AND contraceptive OR contraception” searching for the literature published on any date was also used. Additionally, reference lists of relevant papers were used as an additional source of papers.
